# Permanent stoma after sphincter preservation for rectal cancer. A situation that occurs more often than you might think

**DOI:** 10.3389/fonc.2022.1056314

**Published:** 2023-01-26

**Authors:** Flavie Thomas, Benjamin Menahem, Gil Lebreton, Karine Bouhier-Leporrier, Olivier Dejardin, Arnaud Alves

**Affiliations:** ^1^ Centre Hospitalier Universitaire de Caen, Caen, France; ^2^ Institut Nationale de la Recherche Medicale (INSERM) U1086 Unité de recherche Interdisciplinaire pour la Prévention et le Traitement des Cancers, Caen, France

**Keywords:** rectal cancer, permanent stoma, sphincter preserving surgery, anastomotic leakage, local recurrence

## Abstract

**Objectives:**

This study aimed: (i) to assess the cumulative incidence of permanent stoma (PS) after sphincter-preserving surgery (SPS) for rectal cancer (RC): (ii) to analyze associated risk factors for primary and secondary PS; and (iii) to compare the long-term survival of patients according to the stoma state.

**Methods:**

We conducted a retrospective single-center cohort study based on a prospectively maintained database of SRC patients undergoing SPS from January 2007 to December 2017. Incidence of both primary (no reversal of defunctioning stoma) and secondary (created after closure of defunctioning stoma) PS were investigated. Associations between potential risk factors and PS were analyzed using a logistic regression model. Cumulative survival curve was drawn by Kaplan-Meier method.

**Results:**

Of the 257 eligible patients, 43 patients (16.7%) had a PS (16 primary PS and 27 secondary PS) after a median follow-up of 4.8 years. In multivariate analysis, the independent risk factors for primary PS were severe post-operative complications (OR 3.66; 95% CI, 1.19-11.20, p=0.022), and old age (OR 1.11; 95% CI 1.04-1.18, p=0.001) and those for secondary PS were local recurrence (OR 38.07; 95% CI 11.07-130.9, p<0.0001), anastomotic leakage (OR 7.01; 95% CI, 2.23-22.04, p=0.009), and severe post-operative complications (OR 3.67; 95% CI, 1.22-11.04, p=0.02), respectively. Both overall survival (OS) and disease-free survival (DFS) were significantly lower in patients with a PS compared with patients with SPS (p < 0.01).

**Conclusions:**

This present study suggests that one out of 6 patients has a PS, 5 years after rectal resection with SPS for SRC.

## Introduction

In recent years, progress in the multimodal treatments of rectal cancer (RC) has improved local disease control and increased the survival rate (up to 50% survival at 5 years) (1, 2). At the same time, the evolution of surgical techniques and the achievement of a 1cm distal margin below the tumor have pushed back the limits of sphincter-preserving surgery (SPS) without impairing oncological prognosis (3, 4). Up to 80% of patients with RC undergo SPS with increasingly lower colorectal anastomosis (5). Consequently, a defunctioning stoma is usually created temporarily in both low colorectal (<6cm from the anal margin) and colo-anal anastomosis in order to decrease both morbidity and mortality from anastomotic failure at the time of surgery (6, 7). Despite the intention of SPS, a significant percentage of patients (up to 25%) will live with a permanent stoma (PS) over the long term (8–13). A stoma is commonly considered to be permanent if: (i) it was not closed when it was intended to be temporarily (primary PS); (ii) it was created in a second procedure after the index surgery for any reason, even after the closure of the defunctioning stoma (secondary PS). While the risk of a PS after SPS has been evaluated mainly for non-closure of temporarily stoma (10, 14–18), to our knowledge, a limited number of studies have been devoted to assess the rate for PS of any kind after SPS (9, 19–21). Indeed, the estimation of the risk for PS in long-term follow-up is challenging. Furthermore, several factors of PS has been suggested such as old age, neo-adjuvant radiotherapy, colo-anal anastomosis, anastomotic leakage, poor functional outcome as well as local recurrence but are not well documented (16).

In the present study, we aimed to assess the cumulative incidence of PS after SPS for RC. We also analyzed associated risk factors for primary and secondary PS outcome. Additionally, we compared the long-term survival of patients according to the stoma state.

## Methods

### Patients

All consecutive patients who underwent elective curative surgery for subperitoneal RC (located at or below 10 cm from the anal verge) with SPS between January 2007 and December 2017 in the department of digestive surgery of the university hospital center in Caen were retrospectively identified from the institutional review board-approved prospective database (CNIL: 2204611 v 0) (21). Specifically excluded from this study were patients with intraperitoneal rectal cancer (10cm above the anal verge), chronic inflammatory bowel disease or familial adenomatous polyposis, non-adenocarcinomic rectal cancer, recurrent rectal cancer, and as well as those having undergone either narrow local excision or abdominoperineal excision or Hartmann procedure.

### Data collection

The standardized prospective database included at least patient demographics, ASA classification, body mass index, height of tumor, neoadjuvant and adjuvant therapy, type of operation, TNM (tumor, node, metastasis) staging system, postoperative morbidity and mortality (22).

### Operative procedure and follow-up

Surgical resection was carried out approximatively 8 weeks after the completion of the chemoradiation therapy, when indicated. During the surgical procedure which was previously reported (23), the decision on creating diverting stoma, either loop ileostomy or transverse loop colostomy, was at the discretion of the operating surgeon. Although the decision is left to the surgeon, a loop colostomy rather than a loop ileostomy was performed in elderly patients, patients with limited or impaired renal function, and remote patients, because of the risk of dehydration and ionic disturbances secondary to the ileostomy.

As a general rule, a diverting stoma was made in low colorectal anastomosis (at less that six centimeters from the anal verge) (7). Stoma was closed either 2-3 months after the first operation or at the end of the adjuvant chemotherapy. Before stoma closure, all patients had a digital rectal examination and enema contrast examination to check the integrity of the anastomosis. For the present study, we retrospectively collected the date of stoma reversal, the reasons for not reversing the stoma, the date of a subsequent stoma in a patient whose stoma was not initially a diverting stoma, and finally the date of a repeat stoma procedure after closure of a diverting stoma.

All patients entered a standard clinical, radiological, and endoscopic follow-up program according to French guidelines (3). All patients were followed-up after surgery until death or September 2019. The general practitioner of each patient still alive at the time of collection has been called to obtain the most recent news.

### Study endpoints and outcomes measures

For analysis with regard to stoma rate at the end of follow-up, patients were divided into two groups: those with a PS whether primary or secondary and those with a non-PS after SPS. The primary outcome was to assess the long-term cumulative incidence of a PS regardless of the cause, at the end of follow up (which was calculated from the date of the primary surgery to the date of the last assessment). The secondary outcomes were (i) to identify predictive risk factors associated with primary and secondary PS, and (ii) to compare the long-term survival of patients according to the stoma state.

A PS included primary stoma which was defined as a defunctioning stoma that was not taken down during the study period and secondary stoma which was created after the index surgery or after defunctioning stoma closure, at the endpoint of the study.

Post-operative complications were analyzed according to Dindo-Clavien classification (24); for patients with multiple complications, the highest grade was considered. Anastomotic leakage was defined and graded according to the definition of the International Study Group of Rectal Cancer (25). Anastomotic stricture was defined as non-passage of 13.2-mm diameter colonoscopy and managed with manual and Hegar dilator. Peri-anastomotic complications included anastomotic leakage, pelvic abscess, and anastomotic stricture.

Overall survival (OS) and disease-free survival (DFS) were calculated from the date of surgery to the date of death from any cause or to the date of recurrence, respectively.

### Statistical analysis

Results of descriptive continuous variables are presented as mean ± 1 standard deviation, median with range for quantitative data and numbers with percentage for qualitative data. Dichotomous variables were tested with the Chi-square. The 10% significative variables in the univariate analysis were included in a logistic multivariate analysis with backward procedure, to find independent prognosis factors of recurrence. A result of p<0.05 was considered as statistically significant.

Cumulative survival curve was drawn by Kaplan-Meier method, and each group was comparing using the log-rank test. A result of p<0.05 was considered as statistically significant.

The statistical analyses were performed using SAS 9.4 (SAS Institute Inc., Cary, NC, USA).

## Results

### Patients

**Figure 1:**
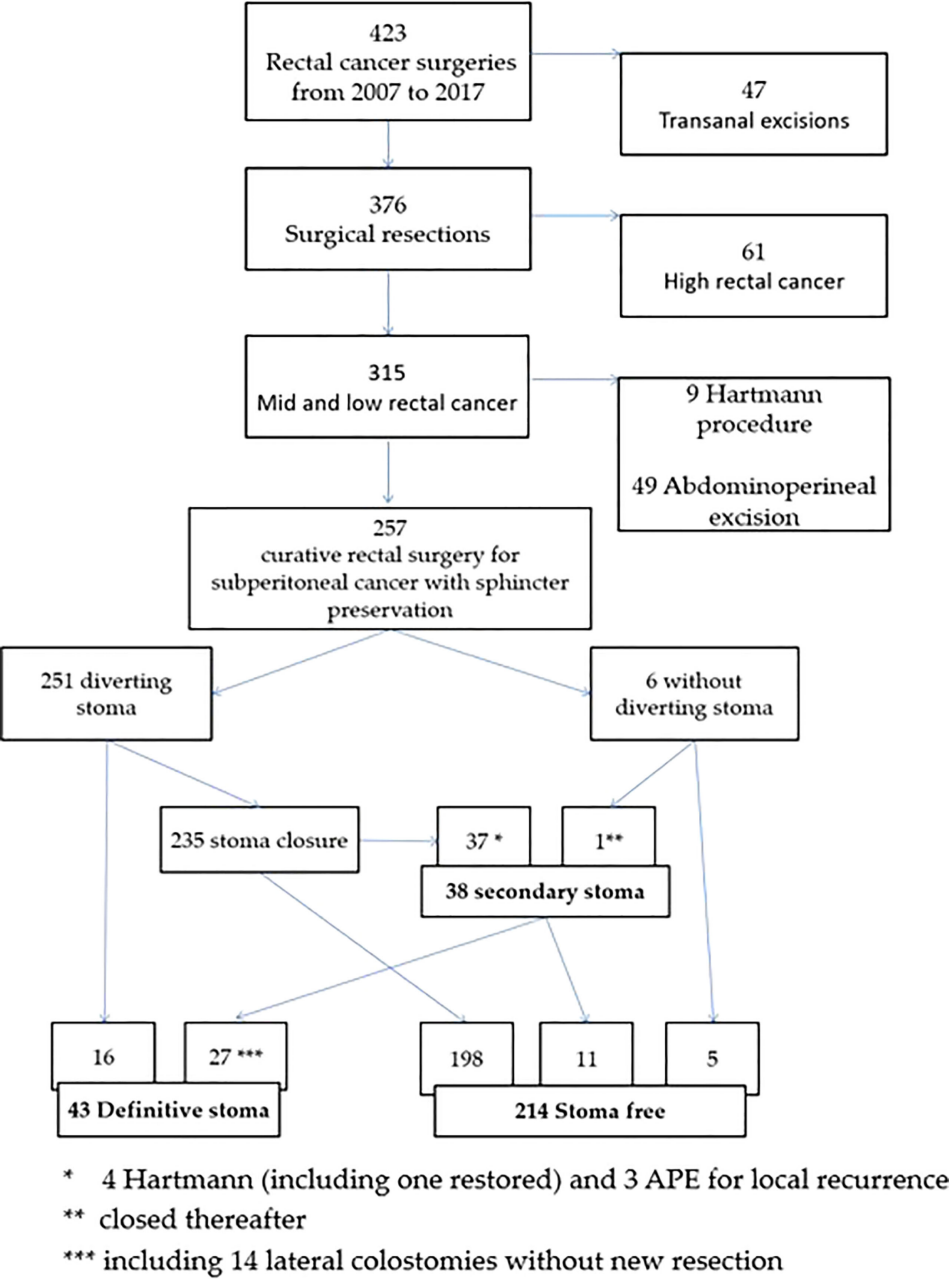
Flow diagram for the study.

Of the 423 patients enrolled, 257 remained eligible following exclusion (**Figure 1**). After a median follow-up of 4.8 years (range 0.02-10.8 years), 43 patients (16.7%) had a PS consisting of 16 patients with primary PS (34%) and 27 patients (66%) with secondary PS (**Figure 1**). Median time to close temporary stoma was significantly shorter for successful SPS patients compared to patients who had re-create PS later (3.46 versus 4.46 months, p=0.005).

Univariate association for a PS are displayed in **Tables 1** and **Tables 3**–**5**. Factors associated with a tendency of PS were history of chronic obstructive pulmonary disease (p=0.003), colo-anal manual anastomosis (p=0.05), severe post-operative complications according to the Dindo-Clavien ≥ 3 (p= 0.0003), anastomotic complications (i.e., deep abscess, anastomotic leakage) (p<0.05), and both local (p<0.0001) and distant recurrence (p=0.0007). Multivariate analysis data for PS are provided also in **Table 1**. The female sex (odds ratio (OR) 2.60; 95% confidence interval (95% CI),1.06-6.40, p= 0.03), history of chronic obstructive pulmonary disease (OR 5.64; 95% CI, 1.48-21.42, p= 0.01), severe post-operative complications according to the Dindo-Clavien ≥ 3 (OR 5.87; 95% CI, 2.32-14.83, p=0.0002), anastomotic leakage (OR 3.52; 95% CI, 1.26-9.80, p=0.01), overall recurrence (OR 2.83; 95% CI, 1.09-7.35, p=0.03), and local recurrence (OR 6.36; 95% CI 1.81-22.32, p<0.003) were independent risk factors for the PS.

**Table 1:** Characteristics of the patients.

		PS N = 43 (%)	SPS N = 214 (%)	P values (CHI2)
Sex	Female	17 (39.53)	87 (40.65)	0.89
	Male	26 (60.47)	127 (59.35)	
Age	Années	67.55 (σ= 11.81)	64.89 (σ= 10.59)	0.32
ASA score	I	5 (11.63)	36 (16.82)	0.20
	II	25 (58.14)	140 (65.42)	
	III	12 (27.91)	37 (17.29)	
	IV	1 (2.33)	1 (0.47)	
Weight (kg)		75.17 (σ= 17.78)	74.04 (σ=17.14)	0.71
BMI> 30		8 (18.60)	33 (15.42)	0.60
Pre-operative weight loss		2.70 (σ=3.55)	1.73 (σ= 4.24)	0.16
Cardiovascular history		19 (44.19)	112 (52.34)	0.32
Neurological history		3 (7.14)	8 (3.79)	0.33
Pulmonary history		8 (18.60)	27 (12.62)	0.29
Chronic obstructive pulmonary disease		7 (16.28)	8 (3.74)	**0.0014**
Previous abdominal surgery		18 (41.86)	95 (44.39)	0.76
Anticoagulant or anti-aggregant		12 (20)	46 (22.01)	0.81

Bold values are for statistically significant p value.

### Stoma outcomes

Of the 257 patients included in this study, 251 (97.6%) had a defunctioning stoma performed at the time of the anterior resection. Of the remaining 6 patients, only one patient experienced an anastomotic leakage that required a secondary stoma. This was closed during the follow-up (**Figure 1**). No patient without a defunctioning stoma was a PS carrier at the end of the study.

Among the 251 patients primarily diverted, the stoma was never closed in 16 of them. The most common reason was anastomotic leakage (31.3%), followed by systemic disease progression (25%) and the patient’s frailty (19%). In multivariate analysis, severe post-operative complications according to the Dindo-Clavien ≥ 3 (OR 3.66; 95% CI, 1.19-11.20, p=0.022), and old age (OR 1.11; 95% CI 1.04-1.18, p=0.001) were independent risk factors for the non-reversal stoma (**Table 2A**).

**Table 2:** Carcinological features.

		PS N = 43 (%)	SPS N = 214 (%)	P values (CHI2)
Location	Mid rectum	26 (60.47)	136 (63.55)	0.70
	Lower rectum	17 (39.53)	78 (36.45)	
Tumour distance to the sphincter		5.71 (σ = 3.69)	5.22 (σ= 3.15)	0.38
Initial stage	0	4 (9.30)	11 (5.14)	0.32
	I	7 (16.28)	31 (14.49)	
	II	12 (27.91)	48 (22.43)	
	III	14 (32.56)	91 (42.52)	
	IV	2 (4.65)	24 (11.21)	
Neoadjuvant therapy		29 (67.44)	161 (75.23)	0.28
Neoadjuvant radiotherapy		29 (67.44)	158 (73.83)	0.39
Inter-sphincteric resection		5 (11.63)	21 (9.81)	0.71
Colorectal anastomosis		9 (20.93)	50 (23.36)	**0.05**
Manual coloanal anastomosis Mechanical coloanal anastomosis		21 (48.84)	65 (30.37)	
		13 (30.23)	99 (46.26)	
Defunctioning stoma		43 (100)	208 (97.20)	0.30
	No	0	6 (2.80)
	Ileostomy	29 (67.44)	162 (75.70)
	Colostomy	13 (30.23)	44 (20.56)
	Ileocolostomy	1 (2.33)	1 (0.93)

Bold values are for statistically significant p value.

The remaining 235 patients received stoma closure. A secondary stoma was required in 38 patients with a median interval of 18.7 months after initial surgery. This was closed only in 11 of them with a mean of 1.72 +/-1.27 surgical procedures per patient. Of the remaining 27 patients, permanent colostomy was the majority in 4 out of 5 patients (81.4%). Three patients underwent an abdominoperineal excision and 3 other patients were treated with a low Hartmann’s procedure (**Figure 1**). The most common indication of secondary PS was local recurrence (29%), uncontrolled pelvic sepsis due to anastomotic leakage (21%), colonic ischeamic stenosis (16%) and J-pouch complications (11%). Severe anorectal dysfunction was associated with both chronic pelvic sepsis and colonic stricture. In multivariate analysis, local recurrence (OR 38.07; 95% CI 11.07-130.9, p<0.0001), anastomotic leakage (OR 7.01; 95% CI, 2.23-22.04, p=0.009), and severe post-operative complications according to the Dindo-Clavien ≥ 3 (OR 3.67; 95% CI, 1.22-11.04, p=0.02) were independent risk factors for secondary PS (**Tables 2B**, **3**).

**Table 3:** Short-term surgical follow-up.

		PS N = 43 (%)	SPS N = 214 (%)	P values (CHI2)
Dindo-Clavien	<3	25 (58.14)	179 (83.64)	**0.0002**
	≥3	18 (41.86)	35 (16.73)	
Deep abscess N=29	Yes	9 (20.93)	20 (9.35)	**0.02**
	No	34 (79.07)	194 (90.65)	
Fistula N= 32	Yes	10 (23.26)	22 (10.28)	**0.01**
	early	5 (11.63)	6 (2.80)	
	delayed	5 (11.63)	16 (7.48)	
	Non	33 (76.74)	192 (89.72)	
Management of fistula/abscess N=53	Medical treatment N=33	7	26	
	Radiological Drainage N=10	2	8	
	Surgical revision N=10	5	5	
Anastomosis stenosis	Yes	8 (18.60)	19 (8.88)	**0.05**
	No	35 (81.40)	195 (91.12)	
Management of stenosis N=27	Manual dilatation	4	16	
	Endoscopic dilatation	2	2	
	Surgical revision	1	0	
	No closure of the defunctioning stoma	1	0	

Bold values are for statistically significant p value.

### Follow-up

Perioperative mortality was 0.3% (1/257) because of myocardial infarction. Only one patient (0.3%) with a defunctioning stoma was lost to follow-up.

**Figure 2:**
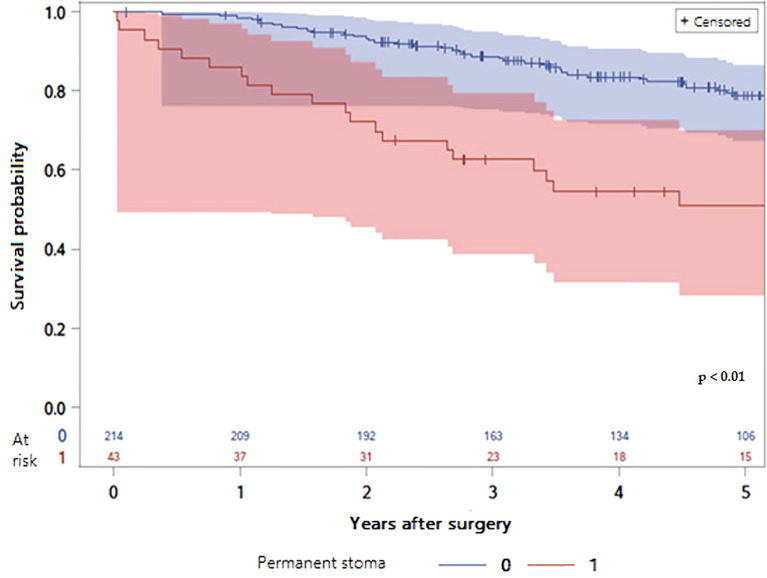
LogRank Test: Comparison of overall survival probabilities according to the presence of a permanent stoma or not with 95% confidence interval (P value < 0.01).

**Figure 3:**
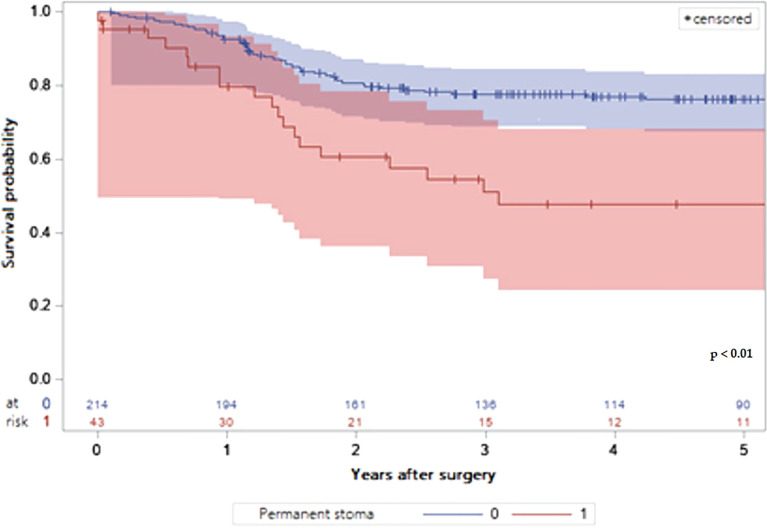
LogRank Test: Comparison of the probabilities of disease free survival depending on the presence of a permanent stoma or not with 95% confidence interval.

The survival data according to stoma states are depicted in **Figures 2**, **3**. Both overall survival (OS) and disease-free survival (DFS) were significantly lower in patients with a PS compared with stoma-free patients (respectively 44.19 versus 74.30% and 53.49 versus 76.17%; P value of log rank test < 0.01) (**Tables 4**, **5**).

**Table 4:** Long-term surgical follow-up.

		PS N = 43 (%)	SPS N = 214 (%)	P values (CHI2)
Adjuvant treatment	Yes	17 (40.48)	78 (36.45)	0.62
	No	25 (59.52)	136 (63.55)	
Ostomy closure time (Average)	If closed	8.20 months (σ=13.13)	5.03 months (σ=3.54)	**0.005**
	If adjuvant treatment	8.29 months (σ= 6.18)	7.70 months (σ=3.90)	**0.005**
	Without adjuvant treatment	8.15 months (σ=15.60)	3.51 months (σ=2.18)	**0.05**
Global recurrence	Yes	20 (46.51)	51 (23.83)	**0.002**
	No	23 (53.49)	163 (76.17)	
Locoregional recurrence	Yes	12 (27.91)	9 (4.21)	**<0.01**
	No	31 (72.09)	205 (95.79)	
Metastatic recurrence	Yes	13 (30.23)	51 (23.94)	0.38

Bold values are for statistically significant p value.

**Table 5:** Univariate and multivariate analyses for risk factors for PS.

				Univariate			Multivariate		
			N	OR	95% CI	P values	OR	95% CI	P values
**Patients**	Sex	Female	104	0.95	0.48 ; 1.86	0.89	**2.60**	1.06 ; 6.40	**0.03**
		Male	153	REF					
	COPD		15	5	1.71 ; 14.66	0.003	5.64	1.48 ; 21.42	**0.01**
**Complications **	DINDO-CLAVIEN	<3	204	REF		0.0003	REF		**0.0002**
		≥3	53	3.68	1.81 ; 7.45		5.87	2.32 ; 14.83	
	Fistula		32	2.64	1.14 ; 6.08	0.02	3.52	1.26 ; 9.80	**0.01**
**Recurrence**	Global recurrence		69	3.34	1.66 ; 6.72	0.0007	2.83	1.09 ; 7.35	**0.03**
	Locoregional recurrence		21	8.81	3.43 ; 22.64	< 0.0001	6.36	1.81 ; 22.32	**0.003**

Bold values are for statistically significant p value.

## Discussion

This present study suggests that one out of 6 patients has a PS, 5 years after rectal resection with SPS for subperitoneal RC. Non reversal of a defunctioning stoma or secondary construction of a stoma remain the two conditions responsible for ending up with a PS. While the risk of a primary PS included older age and severe postoperative complications, local recurrence and anastomotic leakage were the two main factors of secondary PS. Additionally, PS status was significantly associated with a decreased both overall and disease-free survival.

The prevalence of PS (16.7%) reported in the present study is in line with the 3-24% prevalence described in the literature (8–21, 26–31). Unlike most published series, this present study included both primary and secondary PS because their natural histories and risk factors were not the same.

In the present study, non-reversal of a defunctioning stoma affects more than one third of patients with a PS (37.2%). Furthermore, the prevalence of non-reversal of a defunctioning stoma was 6.5% at 4.8 years, with figures in the literature ranging from 3 to 32% after 1.5-7.1 years (8–11, 13, 17, 27). The main risk factors for not being able to reverse a stoma were: older age and severe post-operative complications. These results had already been reported in few studies (12, 13, 20), especially in the Dutch TME trial where the permanent stoma rate reached 19% (10). There are two main reasons for these results. Firstly, surgeons are reluctant to close a defunctioning stoma in case of severe post-operative complications, all the more so in an elderly patient, because of the higher operative risk and the frailty of the patient. Secondly, primarily diverted patients, who are older and/or experienced severe postoperative complications after the index operation are more willing to not only refuse further surgery but also accept a stoma than other patients. Ultimately, the decision not to close a defunctioning stoma most often depends on the surgeon’s and/or the patient’s motivation. This is therefore a key message of the work carried out and a plea for clear, complete and precise information at the preoperative consultation where the stoma is often presented as a temporary phase and where a deadline for closure of the stoma is often announced.

Although, technical advances in colorectal surgery have improved over the two decades, septic post-operative complications (i.e., anastomotic leakage) remains still frequent after low colorectal or coloanal anastomosis. Furthermore, anastomotic leakage remains the main cause of a PS beyond 5 years after colorectal surgery and its severity grade correlated with the PS rate (32). There are currently few data available regarding therapeutic modalities for anastomotic leakage (especially the need for iterative anastomosis creation) in order to preserve a low colorectal or coloanal anastomosis. Recently, Nassar et al. have reported a functional preservation rate of the lower colorectal anastomosis in up to 61% of patients without PS despite an anastomotic leakage (33). In this series including 156 patients with anastomotic leakage, the authors proposed a conservative approach when possible and performed redo surgery for chronic anastomotic failure. Recently, endoluminal vacuum therapy is likely to represent an alternative therapy to iterative surgery in order to preserve low colorectal anastomosis () (34). According to a multicentric study, endoluminal vacuum therapy was effective to treat colorectal anastomotic leakage in more than half of patients, especially when it is used early (within 15 days) and as primary treatment of the anastomotic leakage. Further studies are needed to evaluate long-term functional outcomes of rescue techniques (i.e., endoluminal vaccum therapy or redo surgery for colorectal anastomoses complicated by anastomotic fistulas.

However, events leading to secondary PS are different and were analyzed separately. In the present study, less than two thirds of PS (62.8%) was performed secondarily after closure of the defunctioning stoma. Among patients who required secondary stoma, nearly 3/4 of them (71%) retained the stoma permanently at the end of the study. This study supports that both anastomotic leakage and local RC recurrence were the two independent factors strongly related to the permanent use of stoma, similar to previous studies (10, 11, 13, 19, 29, 31).

Conversely, neither neoadjuvant chemoradiation, nor the advanced T stage, nor the height of the anastomosis did not seem to directly influence the failure of SPS. However, these factors can be considered indirectly involved in the permanent nature of the stoma as they represent risk factors for either anastomotic leakage or local recurrence (35). Moreover, the risk of local recurrence would be increased in the case of an anastomotic leakage (36). It is now reasonable to think that the failure of SPS is probably of multifactorial origin as the literature suggests (35).

Consequently, OS and DFS of the patients with PS were significantly lower than the patients without permanent stoma as some series have already reported (12, 29, 30). These results can be explained by the inherent risk factors for PS. First, older patients have a lower overall survival than younger patients. Second, the occurrence of severe post-operative complications may delay or prohibit adjuvant chemotherapy when indicated, resulting in reduced survival (37, 38). Thirdly, anastomotic leakage was significantly associated with greater local recurrence risk and worse overall and cancer-specific survival (33), although this issue is still debated (39). Finally, the occurrence of local recurrence significantly worsens the prognosis, despite standardized care (40).

The present study has several limitations. First, it was a retrospective analysis of a prospective database, which may have caused biases. Events after stoma closure might be underestimated during postoperative follow-up. Second, we did not evaluate quality of life and postoperative anorectal function systematically. However, the validation of the French-LARS score will allow the use of a scientific instrument to assess both the prevalence and severity of anorectal function (41). Third, this study did not include non-clinical determinants such as social and territorial inequalities (42).

## Conclusion

This study suggests that the proportion of patients with a PS reaches 16.7%, 5 years after removal of subperitoneal RC with SPS. Consequently, patients should be informed of the risk of a PS in planning the surgery either by not closing the temporary stoma, or by re-creating a secondary stoma. In view of these results, other surgical therapeutic alternatives could then be considered, such as abdominoperineal excision or low Hartmann’s procedure. Low hartmann procedure might be a therapeutic alternative to abdominoperineal excision, especially in the co-morbid elderly subject with a potential risk of anal incontinence and in order to avoid both abdominal and perineal postoperative complications. Further studies including digestive function and quality of life are needed to confirm these results.

## Data availability statement

The original contributions presented in the study are included in the article/supplementary material. Further inquiries can be directed to the corresponding author.

## Ethics statement

The studies involving human participants were reviewed and approved by DRCI CHU CAEN. The ethics committee waived the requirement of written informed consent for participation.

## Author contributions

Design of the study: FT, AA, and BM; Registered data: FT; Statistics: BM and OD; Writing manuscript: FT and BM; Revision and supervision: KB-L, GL, and AA. All authors contributed to the article and approved the submitted version.
